# A First-in-Human Randomized, Double-Blind, Placebo-Controlled, Single- and Multiple-Ascending Oral Dose Study of Novel Antimalarial Spiroindolone KAE609 (Cipargamin) To Assess Its Safety, Tolerability, and Pharmacokinetics in Healthy Adult Volunteers

**DOI:** 10.1128/AAC.03393-14

**Published:** 2014-10

**Authors:** F. Joel Leong, Ruobing Li, Jay Prakash Jain, Gilbert Lefèvre, Baldur Magnusson, Thierry T. Diagana, Peter Pertel

**Affiliations:** a Novartis Institute for Tropical Diseases, Singapore; b Novartis Institutes for BioMedical Research, Beijing, People's Republic of China; c Novartis Healthcare Private Limited, Hyderabad, India; d Novartis Pharma AG, Basel, Switzerland; e Novartis Institutes for BioMedical Research, Cambridge, Massachusetts, USA

## Abstract

This first-in-human randomized, double-blind, placebo-controlled, ascending-single and -multiple oral dose study was designed to evaluate the safety, tolerability, and pharmacokinetics in healthy volunteers of KAE609 (cipargamin; formerly NITD609), a spiroindolone now in trials for malaria treatment. It was studied in single-dose cohorts (1 to 300 mg, including one 30-mg food effect cohort) with 4 to 10 subjects in each cohort and in multiple-dose cohorts (10 to 150 mg once daily for 3 days) with 8 subjects in each cohort. The follow-up period was 6 to 8 days post-last dose. Safety and pharmacokinetics were assessed at scheduled time points during the study. Systemic exposure in terms of the area under the concentration-time curve from 0 h extrapolated to infinity (AUC_0–∞_) increased in a dose-proportional manner over the dose range of 1 to 300 mg. The AUC from time zero to the time of the last quantifiable concentration (AUC_last_) and the maximum concentration of drug in plasma (*C*_max_) also increased in an approximately dose-proportional manner. When administered daily for 3 days, the accumulation ratio on day 3 (the AUC from time zero to 24 h postdosing [AUC_0–24_] on day 3/AUC_0–24_ on day 1) was in the range of 1.5 to 2 in the studied dose range (10 to 150 mg) and was consistent with an elimination half-life of around 24 h. Urine analysis for unchanged KAE609 revealed negligible amounts (≤0.01%) were excreted renally. The high fat food intake did not affect the extent of KAE609 absorption (AUC); however, the *C*_max_ was reduced by around 27%. KAE609 was tolerated in this study, with transient gastrointestinal and genitourinary adverse events of mild to moderate intensity (semen discoloration, diarrhea, nausea and abdominal discomfort, dizziness and headache, catheter site hematoma). Gastrointestinal and genitourinary adverse events increased with rising doses.

## INTRODUCTION

Artemisinin derivatives in combination (e.g., Coartem) are currently the treatment of choice for falciparum malaria infections. However, artemisinin resistance has now been documented in Southeast Asia (1–3), and there is an urgent need for new antimalarials to address this threat (4–8). Two new classes of antimalarials have been discovered: the spiroindolones (9, 10), followed by the imidazolopiperazines (11, 12). KAE609 (cipargamin; previously known as NITD609) is a spirotetrahydro-β-carbolines (spiroindolone) and the first new antimalarial chemotype in 2 decades. The spiroindolone class was identified by using high-throughput phenotypic screening (13, 14) and is believed to inhibit PfATP4, a parasite plasma membrane Na+-ATPase, which results in disruption of parasite sodium homeostasis (15). KAE609 has a low nanomolar 50% inhibitory concentration against drug-sensitive Plasmodium strains and is equally potent against various drug-resistant strains. *In vitro* studies indicate activity on both asexual and sexual forms of the parasite, including against stage 5 Plasmodium falciparum gametocytes (10). In an experimental malaria mouse model, KAE609 was more active than artemether, and single-dose cure has been demonstrated in a Plasmodium berghei rodent model of blood-stage malaria (9). This potent antimalarial activity observed in a preclinical rodent model was borne out in a human malaria patient trial, which followed this first-in-human study but was published in advance (16), that showed that KAE609 clears parasitemia rapidly in falciparum and vivax malaria patients.

The objectives of this first-in-human study were to assess the safety and tolerability of KAE609 in healthy adult volunteers after single and multiple oral dosing and to assess the pharmacokinetics after single (including food effect) and multiple dosing.

## MATERIALS AND METHODS

### Study design.

This was a single-center, double-blind, randomized placebo-controlled trial. It was conducted from 16 November 2010 to 4 October 2011 at the Q-Pharm Phase I Unit (Brisbane, Australia). This study comprised two parts.

Part 1 involved single-ascending dose (SAD) cohorts with different dose levels of KAE609 (1 mg, 3 mg, 10 mg, 30 mg, 100 mg, 200 mg, 300 mg) and one food effect cohort (30 mg) with 4 to 10 subjects (including placebo) in each cohort. It consisted of a maximum 28-day screening period, a baseline period (day −1), treatment period (days 1 to 5), and a single study completion evaluation conducted between 6 to 8 days after the dose. The follow-up period was 6 to 8 days postdose.

Part 2 examined multiple-ascending dose (MAD) cohorts administered different dose levels (10 mg, 30 mg, 60 mg, 100 mg, and 150 mg once daily for 3 days) with 8 subjects in each cohort. It comprised a maximum 28-day screening period, a baseline period (day −1), treatment period (days 1 to 7), and a single study completion evaluation conducted on day 9, day 10, and day 11 (which was 6 to 8 days after the last dose). The follow-up period was 6 to 8 days after the last dose.

Study treatment was administered orally with 180 to 350 ml of water, with mouth checks to ensure compliance. All subjects fasted (i.e., no food and liquid except water) for at least 10 h prior to administration of study drug and continued fasting for at least 4 h thereafter. No fluid intake apart from the fluid given at the time of drug intake was allowed from 2 h before until 2 h after dosing. Subjects did not consume any carbonated drinks, and consumption of alcohol, caffeine, tobacco (or nicotine-containing products), or grapefruit (or related citrus fruit) was not permitted during the study. Subjects who were enrolled in the food effect cohort were given the standard FDA breakfast (17) and dosed with KAE609 within 5 min after completion of the meal.

Prescription and nonprescription drugs and dietary supplements were not permitted and had to be discontinued within 30 days or 5 half-lives (whichever was longer) prior to the enrollment. Treatment was to be discontinued in the event of the development of common terminology criteria for adverse events (CTCAE; version 4.0) grade 2 severity (or greater) gastrointestinal, pulmonary, or blood/bone marrow abnormalities or if exposure (i.e., the area under the concentration-time curve from time zero to 24 h postdose [AUC_0–24_]) of KAE609 exceeded 38,800 ng · h/ml, the mean exposure observed in dogs for the highest dose considered safe (5 mg/day for 14 days) in Good Laboratory Practice (GLP) studies. Subjects could be discontinued from the study at their own request for safety, behavioral, or administrative reasons or for any other reason at the discretion of the investigator or sponsor.

This study was conducted in compliance with the Declaration of Helsinki and Good Clinical Practice Guidelines established by the International Conference on Harmonization. The final protocol, amendments, and informed consent documentation were reviewed and approved by the study center's Institutional Review Board. All subjects provided written, informed consent before participating in any study procedures.

### Subjects.

The study planned to enroll 94 subjects (46 subjects in part 1 and 48 subjects in part 2), including both healthy male and female subjects (of non-child-bearing potential), aged 18 to 55 years, of any ethnic origin, of at least 50 kg in weight (with body mass index between 18 and 30 kg/m^2^) who passed screening assessments, met inclusion/exclusion criteria, and provided written consent.

Health status was determined by medical history, physical examination, and laboratory tests at screening. Only females with no child-bearing potential were allowed to enroll, and all female subjects were required to have a negative pregnancy test result at screening and at the baseline. Males were required to comply with barrier contraception methods during the study and up to 3 weeks after the last dose. Exclusion criteria included use of investigational drugs within 30 days or 5 half-lives of enrollment (whichever was greater); history of clinically significant electrocardiogram abnormalities; history of organ system malignancy within the last 5 years (excluding skin basal cell carcinoma); pregnancy or child-bearing potential; use of tobacco products within the previous 3 months; use of prescription medications or herbal supplements within the previous 4 weeks or over-the-counter medication (except for incidental acetaminophen), dietary supplements, or vitamins within 2 weeks; blood donation or blood loss of >400 ml within the previous 8 weeks; and/or a hemoglobin level of <12.0 g/dl. Subjects were also excluded if found to have had significant illness within the previous 2 weeks; history of food allergies or any surgical or medical condition which might significantly alter drug pharmacokinetics; history of immunodeficiency disease; positive hepatitis B or C test result; and/or a history of drug or alcohol abuse in the previous 12 months or evidence thereof.

### Safety assessment.

Safety evaluations included adverse event counts, monitoring for serious adverse events with the severity and relationship to the study drug, monitoring for pregnancies, and regular monitoring of hematology, blood chemistry, urinalysis, vital signs, physical condition, body weight, and electrocardiogram.

### Pharmacokinetic parameters and assessment.

In part 1 of the study (single dosing), blood samples were collected predosing and 0.5, 1, 2, 3, 4, 6, 8, 12, 24, 36, 48, 72, and 96 h postdose. Urine samples (10-ml aliquots) were collected from the fasting cohorts that received a single dose of ≥30 mg, only at predose, and at the end of each of the following periods: 0 to 12 h postdose, 12 to 24 h postdose, and 24 to 48 h postdose.

In part 2 (daily dosing for 3 days), blood sampling on day 1 occurred at predose and 0.5, 1, 2, 3, 4, 6, 8, 12, and 24 h postdose. On day 3, blood samples were collected at predose and 0.5, 1, 2, 3, 4, 6, 8, 12, 24, 36, 48, 72, 96, 120, and 144 h postdose.

For bioanalysis, plasma sample preparation consisted of protein precipitation, evaporation of the filtrate, and analysis of the reconstituted sample. A 10-μl aliquot of the reconstituted extract was analyzed by liquid chromatography-tandem mass spectrometry (LC-MS/MS) in multiple reaction monitoring (MRM) mode using electrospray ionization (ESI) as the ionization technique. KAE609[M + 6] was used as the internal standard. The lower and upper limits of quantification were 1.00 ng/ml and 5,000 ng/ml, respectively, when we used 0.0500 ml of plasma. KAE609 is stable in human plasma for at least 24 h at room temperature, after 3 freeze-thaw cycles, and for at least 322 days after storage at or below −60°C. The coefficient of variation (CV; precision) and percent bias (accuracy) for the multiple runs during the period of analysis for five quality control samples of 2.00, 5.00, 400, 2,000, and 4,000 ng/ml were 9.8 and 2.0, 4.9 and −2.4, 6.1 and −5.3, and 6.8 and 2.0, respectively.

The urine bioanalysis method consisted of sample dilution with an internal standard and analysis of the aliquot by LC-MS/MS in the MRM mode by using ESI as the ionization technique. The lower and upper limits of quantification were 1.00 ng/ml and 5,000 ng/ml using 0.0500 ml of urine. The CV and bias for the multiple runs during the period of analysis for six quality control samples of 1.00, 2.00, 5.00, 400, 2,000, and 4,000 ng/ml were 14.1 and 12.0, 4.3 and 10.0, 6.0 and 17.6, 5.1 and −5.5, 5.4 and 17.5, and 6.8 and 2.0, respectively.

Pharmacokinetic parameters were derived by using a noncompartmental method within WinNonlin Pro (version 5.2). The following pharmacokinetic parameters were derived for part 1: the AUC_0–24_; AUC from time zero to the time of the last quantifiable concentration [AUC_last_; (mass × time)/(volume)]; AUC from time zero extrapolated to infinity (AUC_0–∞_);maximum observed concentration (*C*_max_); elimination half-life (*t*_½_); apparent oral clearance (CL/F); apparent volume of distribution during the terminal elimination phase following extravascular administration (*V_z_*/F). For part 2, the following pharmocokinetic values were determined: AUC_0–24_, *C*_max_, *T*_max_ (for day 1 and day 3), *t*_½_ (post-day 3 dosing), and the accumulation ratio [*R*_acc_; (AUC_0–24_ on day 3/AUC_0–24_ on day 1)]. In addition, the amount of drug excreted into the urine from time zero to time *t* (Ae_0–*t*_) and renal clearance were determined for part 1 from the urine drug concentration-time data collected from subjects who received 30-mg, 100-mg, or 200-mg doses.

### Statistical methods.

Pharmacokinetic parameters were summarized using arithmetic means and standard deviations, except for *T*_max_, for which median values and ranges are reported. To test dose proportionality, the primary pharmacokinetic parameters were analyzed by using a regression model on the log-transformed pharmacokinetic parameters (AUC_last_ and/or AUC_0–∞_ and *C*_max_) versus log-transformed dose, and dose proportionality was assessed with a lack-of-fit test. The analyses were done separately for the single- and multiple-dose cohorts and separately on days 1 and 3 for the multiple-dose cohorts. In the food effect cohort, the geometric mean ratio of primary pharmacokinetic parameters under fed and fasted conditions are reported along with 90% confidence intervals (CI), which were determined from a mixed linear model with fixed effects for fasted/fed condition and a random subject effect.

Adverse events were summarized by treatment group, preferred term, and severity, with formal statistical analysis performed if a dose-related trend was apparent.

## RESULTS

### Subject demographics.

A total of 95 subjects were enrolled and analyzed (46 subjects in part 1 and 49 subjects in part 2), and 93 subjects completed the study. In study part 1 (single dosing), one subject who completed the treatment and assessments in the 30-mg single-dose fasted cohort was found to have an elevated total bilirubin level (26 μmol/liter) when he returned for his second baseline entry criteria check for the food effect cohort (30-mg single dose, fed) and was consequently excluded and withdrawn, resulting in only 34 of 35 subjects in the pooled group completing the study. In study part 2, there was one subject who did not complete the study due to withdrawal of informed consent on day 1 after administration of the first dose and assessment, resulting in only 45 of 46 subjects in the pooled group completing the study. This subject did not have any clinically significant abnormalities recorded. All subjects were male, and the majority were white (94.68%). The demographics and other baseline characteristics are shown in Table 1.

**TABLE 1:** Demographics and other baseline characteristics

Parameter	Part 1			Part 2		
	KAE609 (*n* = 35)	Placebo (*n* = 11)	Total (*n* = 46)	KAE609 (*n* = 37)	Placebo (*n* = 12)	Total (*n* = 49)
Mean age, yrs (range)	25.2 (18–45)	24.6 (18–32)	25.1 (18–45)	25.4 (18–47)	23.1 (20–29)	24.8 (18–47)
No. (%) of:						
Caucasians	33 (94.3)	11 (100)	44 (95.7)	33 (89.2)	12 (100)	45 (91.8)
Asians	1 (2.9)	0 (0.0)	1 (2.2)	4 (10.8)	0 (0.0)	4 (8.2)
Others	1 (2.9)	0 (0.0)	1 (2.2)	0 (0.0)	0 (0.0)	0 (0.0)
Mean wt, kg (range)	76.2 (51–98)	79.2 (58–105)	76.7 (51–105)	76.7 (54–93)	77.0 (70–91)	76.7 (54–93)
Mean ht, cm (range)	179.6 (166–194)	178.0 (164–187)	179.2 (164–194)	179.7 (163–193)	180.3 (171–192)	179.9 (163–193)

### Safety and tolerability.

All 95 randomized subjects were included in the safety analysis. There were no deaths or serious adverse events. The overall incidence of adverse events was higher in subjects administered KAE609 than those given placebo, with the incidence increasing with dose. More subjects experienced adverse events in the multiple-dosing cohorts than single-dosing cohorts (73% versus 50%), and proportionally more of these were considered drug related (62.2% versus 19%). Gastrointestinal events were more frequent in subjects who received KAE609. All events were mild or moderate in severity, with most assessed as mild and resolving spontaneously.

After single doses, 50.0% (21/42) and 15.4% (2/13) of subjects administered KAE609 and placebo, respectively, developed one or more adverse events. Five events occurred in 2 or more subjects administered any dose of KAE609 or placebo. Four events only developed in subjects receiving KAE609: semen discoloration (16.7%), diarrhea (7.1%), nausea (7.1%), and fatigue (4.8%). Headache was reported in subjects administered KAE609 (11.9%) or placebo (7.7%). After multiple doses, 73.0% (27/37) and 50.0% (6/12) of subjects administered KAE609 and placebo, respectively, developed one or more adverse events. Seven events occurred in 2 or more subjects administered any dose of KAE609 or placebo. Four events only developed in subjects who received KAE609: dizziness (10.8%), catheter site hematoma (8.1%), back pain (5.4%), and nausea (5.4%). Three events were reported in subjects administered KAE609 or placebo, including headache (45.9% versus 33.3%, respectively), semen discoloration (59.5% versus 8.3%), and contusion (2.7% versus 16.7%). The events more often assessed as related to the study drug were semen discoloration and headache, with this assessment made among subjects administered KAE609 and those administered placebo. There were no clinically significant abnormal blood pressure measurements, and no hypotension or vomiting was associated with the reported dizziness.

One subject who received multiple doses of KAE609 developed elevated blood alanine transaminase (ALT), aspartate transaminase (AST), lactate dehydrogenase (LDH), and creatine kinase (CK) levels that were assessed as mild adverse events, but these were not attributed to the study drug. His bilirubin level was within the normal range and liver enzyme abnormalities resolved within 4 days. One subject who received multiple doses of placebo developed elevated blood amylase and lipase levels that were assessed as mild adverse events; both were suspected to be related to the study drug.

Vital sign measurements and ECG measurements did not reveal any abnormalities determined to be clinically significant by the investigator in any subject. Mean and median values for measured hematology and blood chemistry parameters (including ALT, AST, LDH, and bilirubin) remained within the normal range from baseline to the end of the study, with the exception of two elevated findings (AST and ALT) at the end-of-study visit in the 100-mg once-daily (q.d.) 3-day cohort; these findings were solely attributable to the subject previously described. There were sporadic individual hematology, chemistry, or urinalysis parameters that were outside the normal range during the study. These were classified by the investigator as not clinically significant or not drug related.

There were 7 subjects in study part 1 and 22 subjects in study part 2 who reported yellow semen discoloration after being dosed. This occurred at different dose levels: 1 subject on the single 200-mg dose, 6 subjects on the 300-mg single dose, 5 subjects on the 60-mg daily dose for 3 days, 11 subjects on the 100-mg daily dose for 3 days, and 6 subjects on the 150-mg daily dose for 3 days. In total, this affected 29/79 (36.7%) subjects who were taking the active drug. Yellow discoloration of semen was confirmed in laboratory examination of collected semen samples, which were voluntarily provided by most subjects after this adverse event was reported. There was no azospermia, diminished motility, or clinically significant abnormalities reported.

Upon further investigation conducted after this event, no KAE609 was detected in ejaculate. However, three primary metabolites of KAE609 (M18, M23, and M48) were present, two of which (M18 and M23) are known to be orange-yellow in color. Of the two metabolites detected, the M23 color was demonstrated in the laboratory at or below concentrations detected in semen. We inferred that the color seen was due to metabolite M23. The main metabolite (M23) is not active against Plasmodium falciparum and shows no cytotoxic potential against a human hepatic cell line (unpublished data). Other minor metabolites have not been tested against P. falciparum but are not believed to be toxic at the levels detected based on assessment of semen samples.

### Pharmacokinetics. (i) Single-dose administration in healthy volunteers (1 to 300 mg, oral administration).

**FIG 1:**
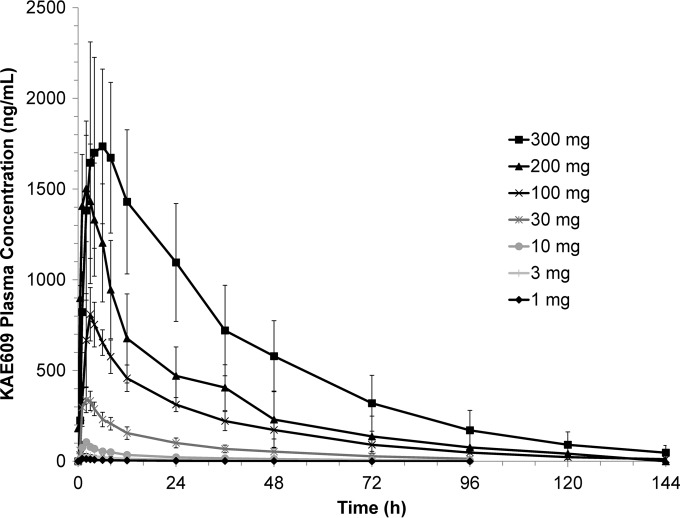
Arithmetic mean linear plasma drug concentration-time profiles for KAE609 following oral administration of single ascending doses (SAD, part 1). Error bars represent ±1SD.

The mean plasma drug concentration-time profile of KAE609 following oral administration of a 1- to 300-mg dose is presented in Fig. 1. The pharmacokinetic parameters are summarized in Table 2. *C*_max_ was in the range of 14 ng/ml (for the 1-mg dose) to 2,090 ng/ml (for the 300-mg dose), with a median *T*_max_ in the range of 1 to 5 h. A trend for an increasing *T*_max_ with increasing dose has been observed, and at the highest dose of 300 mg, the *T*_max_ was in the range of 3 to 8.15 h. The AUC_last_ was in the range of 0.17 h · μg/ml to 79.7 h · μg/ml over the 1- to 300-mg dose range. Systemic exposure in terms of the AUC_0–∞_ (slope, 1.04; 90% CI, 0.99 to 1.09) increased in a dose-proportional manner over the dose range of 1 to 300 mg of KAE609. The AUC_last_ (slope, 1.07; 90% CI, 1.03 to 1.12) and *C*_max_ (slope, 0.89; 90% CI, 0.85 to 0.92) also increased in an approximately dose-proportional manner. The *C*_max_ exhibited better proportionality in the dose range of 1 to 200 mg; however, at 300 mg it was slightly less than proportional, most probably due to the low aqueous solubility of KAE609 (pBCS class II). KAE609 was eliminated with a terminal elimination half-life (*T*_½_) in the range of 19 to 26 h over the 1- to 300-mg dose range in healthy subjects. The apparent volume of distribution and apparent clearance ranged between 126 to 194 liters and 4 to 5.5 liters/h, respectively, over the entire dose range.

**TABLE 2:** Summary of pharmacokinetic parameters of KAE609 following oral administration of single ascending doses (part 1)

Pharmacokinetic parameter	Mean ±SD for dose group						
	1 mg (*n* = 3)	3 mg (*n* = 3)	10 mg (*n* = 3)	30 mg (*n* = 8)	100 mg (*n* = 6)	200 mg (*n* = 6)	300 mg (*n* = 5)
AUC_0–24_ (μg · h/ml)	0.127 ± 0.044	0.388 ± 0.126	1.02 ± 0.092	4.29 ± 0.725	11.5 ± 1.51	23.7 ± 4.99	36.0 ± 2.40
AUC_last_ (μg · h/ml)	0.169 ± 0.054	0.609 ± 0.25	1.75 ± 0.28	7.48 ± 1.98	23.1 ± 4.01	51.8 ± 16.0	79.7 ± 15.8
AUC_0–∞_ (μg · h/ml)	0.217^*a*^	0.66 ± 0.25	1.86 ± 0.321	8 ± 2.47	23.5 ± 4.23	53.7 ± 17.8	81.3 ± 17.7
*C*_max_ (ng/ml)	14.1 ± 3.50	35.4 ± 7.97	105 ± 6.95	364 ± 57.6	830 ± 141	1640 ± 305	2090 ± 482
*T*_max_ (h)^*b*^	1 (1.0–1.0)	2 (2.0–2.0)	2 (2.0–2.02)	2.52 (1.0–3.02)	3 (2.0–4.0)	3.51 (2.0–6.0)	5.43 (3.0–8.15)
*T*_1/2_ (h)	19.4 ± 4.32	23.5 ± 2.65	24.8 ± 2.46	23.2 ± 5.05	22.2 ± 4.97	25.6 ± 9.07	24.0 ± 7.59
CL/F (liters/h)	5.18 ± 1.17	4.92 ± 1.51	5.49 ± 0.99	4.03 ± 1.07	4.40 ± 0.93	4.08 ± 1.33	3.85 ± 1.0
*V_z_*/F (liters)	149 ± 61.1	169 ± 62.3	194 ± 16.9	129 ± 24.0	138 ± 24.8	141 ± 39.0	124 ± 17.7

a Determined for only two subjects.

b *T*_max_ values are medians (with ranges shown in parentheses).

### (ii) Multiple-dose administration in healthy volunteers (10 to 150 mg q.d. for 3 days, oral administration).

**FIG 2:**
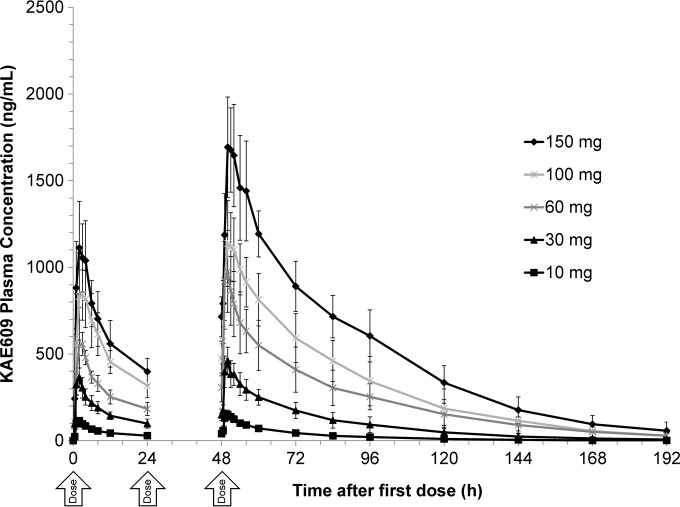
Arithmetic mean day 1 and day 3 superimposed plasma drug concentration-time profiles following oral administration of KAE609 in multiple ascending doses (10, 30, 60, 100, and 150 mg; MAD, part 2). Error bars represent ±1SD.

The day 1 and day 3 superimposed mean plasma drug concentration-time profiles of individual dose levels (10, 30, 60, 100, and 150 mg daily for 3 days) following oral administration of KAE609 are presented in Fig. 2. The pharmacokinetic parameters are summarized in Table 3. The mean *C*_max_ and AUC_0–24_ on day 1 were in the range of 120 to 1,170 ng/ml and 1.24 to 15.1 μg · h/ml, respectively. The corresponding values for day 3 were 161 to 1,770 ng/ml and 1.91 to 29.4 μg · h/ml, respectively. Over the complete dose range, the median *T*_max_ ranged from around 1.5 to 3 h. Systemic exposure in terms of the AUC_last_ on day 3 (slope, 1.00; 90% CI, 0.94 to 1.07) increased in a dose-proportional manner over the dose range of 10 to 150 mg of KAE609. The day 3 *C*_max_ (slope, 0.86; 90% CI, 0.80 to 0.93) also increased in an approximately dose-proportional manner but was not considered statistically dose proportional.

**TABLE 3:** Summary of pharmacokinetic parameters of KAE609 following oral administration of multiple ascending doses (part 2)

Pharmacokinetic parameter	Mean ±SD for dose group on indicated day									
	10 mg		30 mg		60 mg		100 mg		150 mg	
	Day 1	Day 3	Day 1	Day 3	Day 1	Day 3	Day 1	Day 3	Day 1	Day 3
AUC_0–24_ (μg · h/ml)	1.24 ± 0.20	1.91 ± 0.4	4.08 ± 0.72	6.42 ± 1.22	6.99 ± 0.97	14.1 ± 3.48	12.2 ± 1.94	19.8 ± 3.56	15.1 ± 2.73	29.4 ± 4.12
*C*_max_ (ng/ml)	120 ± 16.7	161 ± 29.3	364 ± 55.4	468 ± 69.2	641 ± 91.7	991 ± 216	921 ± 164	1,170 ± 206	1,170 ± 260	1,770 ± 232
*T*_max_^*a*^ (h)	2 (1.0–3.0)	2 (1.0–2.0)	2 (2.0–2.02)	2 (1.0—2.0)	2 (1.0–4.0)	1.5 (1.0–2.0)	3 (1.0–4.0)	2 (2.0–4.0)	2 (2.0–4.0)	2.5 (2.0–8.15)
*T*_1/2_ (h)	NA^*b*^	21.4 ± 4.4	NA	22.4 ± 5.0	NA	28.0 ± 4.5	NA	23.1 ± 6.1	NA	26.9 ± 8.3
R_acc_	1.54		1.57		2.01		1.67		1.98	

a Values are means ± standard deviations, except for *T*_max_ values, which are medians (with ranges shown in parentheses), and R_acc_ values (for which only the means are reported). *N* = 6 to 7 subjects per cohort.

b NA, not available; the *T*_1/2_ was determined on day 3 only.

The elimination half-life was not calculated on day 1 because of the q.d. dosing of KAE609. The elimination half-life was in the range of 21 to 28 h over the 1- to 150-mg dose range. The accumulation index [R_acc_; (AUC_0–24_ on day 3/AUC_0–24_ on day1)] was found to be in the range of 1.54 to 2.01, as expected due to the long (∼24-h) elimination half-life and q.d. dosing.

### Food effect.

The food effect (determined using a high-fat standard FDA breakfast) on the pharmacokinetics of KAE609 was evaluated with a 30-mg single dose. In a comparison of primary pharmacokinetic parameters under fast and fed conditions (30 mg single dose), the extent of absorption was not affected significantly by food intake. The geometric mean fed/fast ratio for the AUC_last_ and AUC_0–∞_ were 1.063 (90% CI, 0.979 to 1.153) and 1.016 (90% CI, 0.947 to 1.091), respectively. Conversely, the mean *C*_max_ under fed conditions (254 ng/ml) was reduced by 27% (geometric mean fed/fast ratio, 0.706) compared to fasting condition (356 ng/ml). The *T*_max_ was delayed from a median of 2.52 h to 4 h under fed conditions. There was no major impact of food on clearance (CL/F; 4.00 liters/h for fed versus 4.03 liters/h for fasted) and volume of distribution (*Vz*/F; 129.36 liters for fed versus 123.46 liters for fasted).

### Exploratory urinary excretion.

KAE609 is minimally excreted via urine in unchanged form and, on average, less than or equal to 0.01% of the administered dose of KAE609 was excreted unchanged in urine over a period of 48 h postdose. The renal clearance (CL_r_) was in the range of 0.438 ml/h to 0.475 ml/h, which was low compared with overall clearance, which was in the range of 4 to 5.5 liters/h. Consequently, urine samples from other cohorts were not analyzed for unchanged drug.

## DISCUSSION

KAE609 was well tolerated in this group of healthy adults, with essentially transient gastrointestinal and genitourinary adverse events of mild to moderate intensity (semen discoloration, diarrhea, nausea and abdominal discomfort, dizziness and headache, catheter site hematoma). Gastrointestinal and genitourinary adverse events increased with rising doses.

The yellow discoloration of semen was an unexpected finding and appears to be an isolated observation attributable to one or more colored metabolites. The presence of drug or drug metabolites in semen is not unusual; caffeine (18), aspirin (19, 20), and chloroquine (21, 22) are some examples of compounds for which this is reported. Where samples were provided, we found no azospermia, diminished motility, or clinically significant abnormality in semen.

Our results demonstrated pharmacokinetic approximate-dose proportionality with minimal food effect. Systemic exposure in terms of the AUC_inf_ (slope, 1.04; 90% CI, 0.99 to 1.09) increased in a dose-proportional manner over the dose range of 1 to 300 mg of KAE609. The AUC_last_ (slope, 1.07; 90% CI, 1.03 to 1.12) and *C*_max_ (slope, 0.89; 90% CI, 0.85 to 0.92) also increased in an approximately dose-proportional manner; however, because the confidence intervals for the slope estimates did not include 1, these were not considered statistically dose proportional. The accumulation ratio on day 3 (AUC_0–24_ on day 3/AUC_0–24_ on day 1) in MAD was in the range of 1.5 to 2 for the studied dose range (10 to 150 mg). The accumulation ratio was consistent with an elimination half-life of around 24 h. No absolute bioavailability studies in humans have been conducted so far.

At a single dose of 30 mg, the extent of absorption (AUC) was not affected by food intake. However, a reduced *C*_max_ (∼27%) and increased *T*_max_ (from 2.5 to 4 h) were observed. This was not felt to be clinically relevant, because the efficacy likely depends on maintaining adequate KAE609 concentrations throughout the dosing period and not achieving specific *C*_max_ levels. Urine analysis for unchanged KAE609 revealed that a negligible amount (≤0.01%) was excreted renally. Similar to many antimalarials, KAE609 is predominantly metabolized in the liver by cytochrome P450 3A4, the clinical relevance of which, in terms of drug-drug interactions, is presently unknown.

There were no findings in this study which would preclude administering this compound to malaria patients. The results of preclinical studies suggest that antimalarial efficacy of KAE609 may be AUC driven. (S. B. Lakshminarayana, C. Freymond, C. Fischli, et al., submitted for publication). Based on the P. berghei mouse model, the daily dose sufficient to clear 99% of the parasites detected in the blood was 5.3 mg/kg; exposure (AUC_0–24_) at this dose was estimated to be 3,260 ng · h/ml based on modeling of pharmacokinetic data obtained with noninfected mice. Allometric scaling of mouse, rat, and dog pharmacokinetic data yielded a predicted equivalent human efficacious dose of 30 mg (predicted AUC_0–24_ of 3,570 ng · h/ml) for a 70-kg human. The healthy volunteer pharmacokinetic data reported here are consistent with these predictions and suggest that a 30-mg daily dose might be sufficient to generate the maximum parasitemia reduction in malaria patients. Indeed, a study which followed this study demonstrated positive results in 2013 (16): a 3-day regimen of 30 mg daily cleared parasitemia in P. vivax and P. falciparum malaria patients.
